# De-ubiquitinating enzyme, USP11, promotes transforming growth factor *β*-1 signaling through stabilization of transforming growth factor *β* receptor II

**DOI:** 10.1038/cddis.2016.371

**Published:** 2016-11-17

**Authors:** A M Jacko, L Nan, S Li, J Tan, J Zhao, D J Kass, Y Zhao

**Affiliations:** 1 Department of Medicine, University of Pittsburgh, Pittsburgh, PA, USA; 2 Department of Anesthesia, The First Affiliated Hospital of Jilin University, Changchun, China; 3 Department of General Surgery, The First Affiliated Hospital of Dalian Medical University, Dalian, China

## Abstract

The transforming growth factor *β*-1 (TGF*β*-1) signaling pathway plays a central role in the pathogenesis of pulmonary fibrosis. Two TGF*β*-1 receptors, T*β*RI and T*β*RII, mediate this pathway. T*β*RI protein stability, as mediated by the ubiquitin/de-ubiquitination system, has been well studied; however, the molecular regulation of T*β*RII still remains unclear. Here we reveal that a de-ubiquitinating enzyme, USP11, promotes TGF*β*-1 signaling through de-ubiquitination and stabilization of T*β*RII. We elucidate the role that mitoxantrone (MTX), an USP11 inhibitor, has in the attenuation of TGF*β*-1 signaling. Inhibition or downregulation of USP11 results in increases in T*β*RII ubiquitination and reduction of T*β*RII stability. Subsequently, TGF*β*-1 signaling is greatly attenuated, as shown by the decreases in phosphorylation of SMAD2/3 levels as well as that of fibronectin (FN) and smooth muscle actin (SMA). Overexpression of USP11 reduces T*β*RII ubiquitination and increases T*β*RII stabilization, thereby elevating phosphorylation of SMAD2/3 and the ultimate expression of FN and SMA. Further, elevated expression of USP11 and T*β*RII were detected in lung tissues from bleomycin-challenged mice and IPF patients. Therefore, USP11 may contribute to the pathogenesis of pulmonary fibrosis by stabilization of T*β*RII and promotion of TGF*β*-1 signaling. This study provides mechanistic evidence for development of USP11 inhibitors as potential antifibrotic drugs for pulmonary fibrosis.

Transforming growth factor *β*-1 (TGF*β*-1) signaling pathway is initiated by the binding of the TGF*β*-1 to TGF*β*-1 receptor II (T*β*RII), which then binds to T*β*RI and activates T*β*RI by phosphorylating a characteristic SGSGSG sequence, called the GS domain.^1, 2^ This phosphorylation signal is then transduced within the cells via SMAD proteins 2 and 3 (SMAD2/3). These SMADS then form a complex with SMAD4, which ultimately translocates to the nucleus where interaction with other transcription factors results in the specific expression of upwards of 500 genes.^3, 4, 5^ Expression of these genes, including fibronectin (FN) 1 and *α*-smooth muscle actin (SMA, gene ACTA2), are associated with the myofibroblast phenotype and progression of fibrosis.^6, 7^ The regulation of T*β*RI and its role in fibrosis have been well elucidated.^8, 9, 10, 11^ Comparatively less is known about its counterpart, T*β*RII.

Regulation of the TGF*β*-1 pathway can be achieved in a number of ways, ubiquitination and de-ubiquitination being key regulators. Ubiquitin is an 8-kDa protein that can be attached to a protein's lysine residue via a three-step process: activation, conjugation, and ligation; each of which are carried out by their own enzyme: E1 ubiquitin-activating enzyme, E2-conjugating enzyme, and E3 ubiquitin ligase, respectively.^12, 13, 14^ Additional ubiquitins can be added to the preceding ubiquitin resulting in a poly-ubiquitinated protein. The number and linkage type of these ubiquitins determine a protein's fate within the cell. The process of ubiquitination can be reversed via a process called de-ubiquitination, which is carried out by de-ubiquitinating enzymes (DUBs).^15, 16, 17^ This process adds another layer to the protein-regulatory processes carried out by the cells. De-ubiquitination by DUBs promotes target proteins' lifespan and regulates protein localization and biological activity.^17, 18, 19^

There are numerous studies on DUBs specific for T*β*RI. It has been revealed that the DUBs, UCH37, USP11, and USP15, de-ubiquitinate and stabilize T*β*RI.^20, 21, 22^ However, very little research has been performed to examine the regulation of its co-receptor, T*β*RII. It is the goal of this study to elucidate the role that DUB USP11 has in the regulation of T*β*RII stability and how the anticancer drug, mitoxantrone (MTX), an inhibitor of USP11,^23^ mitigates TGF*β*-1-induced signaling through reduction of T*β*RII stability.

## Results

### MTX inhibits TGFβ-1-induced phosphorylation of SMAD2/3 and TβRI as well as the expression of FN and SMA in lung fibroblast cells

**Figure 1:**
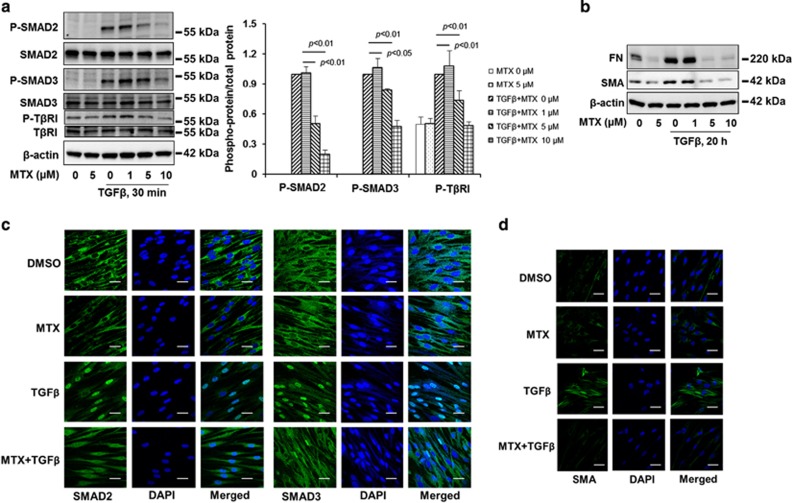
MTX attenuates TGF*β*-1 signaling in human lung fibroblast. (**a**) MRC5 cells were treated with increasing doses of MTX (0, 1, 5, 10 *μ*M) for 1 h, and then cells were treated with TGF*β*-1 (2 ng/ml) for 30 min; the phosphorylated and total forms of SMAD2, SMAD3, and T*β*RI were then analyzed by western blotting. Western blotting images were cropped to improve the conciseness of the data; samples derived from the same experiment and the blots were processed in parallel. Representative of experiments performed at least three independent times. Intensities of blots were measured. The ratio of phosphorylated/total protein was analyzed by the ImageJ software. (**b**) MRC5 cells were treated with increasing doses of MTX (0, 1, 5, 10 *μ*M) for 1 h, and then cells were treated with TGF*β*-1 (2 ng/ml) for 20 h. FN and SMA levels were analyzed by western blotting. Western blotting images were cropped to improve the conciseness of the data; samples derived from the same experiment and the blots were processed in parallel. Representative of experiments performed at least three independent times. (**c**) MRC5 cells grown on glass-bottom dishes were treated with dimethyl sulfoxide (DMSO) or MTX (5 *μ*M) for 1 h, and then cells were treated with TGF*β*-1 (2 ng/ml) for 1 h. Localization of SMAD2 or SMAD3 (green) in the cells were detected by immunostaining with SMAD2 and SMAD3 antibodies. DAPI (4,6-diamidino-2-phenylindole) was used for nuclei staining (blue). Bars, 10 *μ*M. Representative images were shown. (**d**) MRC5 cells grown on glass-bottom dishes were treated with DMSO or MTX (5 *μ*M) for 1 h, and then cells were treated with TGF*β*-1 (2 ng/ml) for 20 h. Localization of SMA (green) in the cells were detected by immunostaining with an SMA antibody. DAPI was used for nuclei staining (blue). Bars, 10 *μ*m. Representative images are shown

MTX, an anticancer drug, has been known to inhibit TGF*β*1-induced COL1A1 expression in human dermal fibroblast cells, suggesting that MTX has an antifibrotic effect.^24^ To further understand the molecular mechanism by which MTX inhibits TGF*β*-1-mediated pro-fibrotic responses, we first examined the effect of MTX on TGF*β*-1 signaling. MRC5 cells were treated with TGF*β*-1 (2 ng/ml) for 30 min, and as expected, phosphorylation of SMAD2/3 (p-SMAD2/3) and T*β*RI (p-T*β*RI) were induced while total SMAD2/3 and T*β*RI levels remained constant (Figure 1a). Ultimately, the levels of FN and *α*-SMA were increased in cells treated with 2 ng/ml TGF*β*-1 for 20 h (Figure 1b). These results were attenuated, however, when the cells were pretreated with MTX. The levels of p-SMAD2/3, p- T*β*RI, FN, and SMA were decreased in a dose-dependent manner (Figures 1a and b). Phosphorylation promotes interaction of SMAD2/3 with SMAD4, leading to nuclear translocation and transcription of FN and SMA. We show that MTX attenuated TGF*β*-1-induced SMAD2 and SMAD3 nuclear translocation (Figure 1c) and expression of SMA (Figure 1d). These data suggest that MTX exhibits an antifibrotic effect and mitigates TGF*β*-1 signaling in the upstream of SMAD2/3 and T*β*RI.

### USP11 promotes TGFβ-1-induced phosphorylation of SMAD2/3 and the expression of FN and SMA

**Figure 2:**
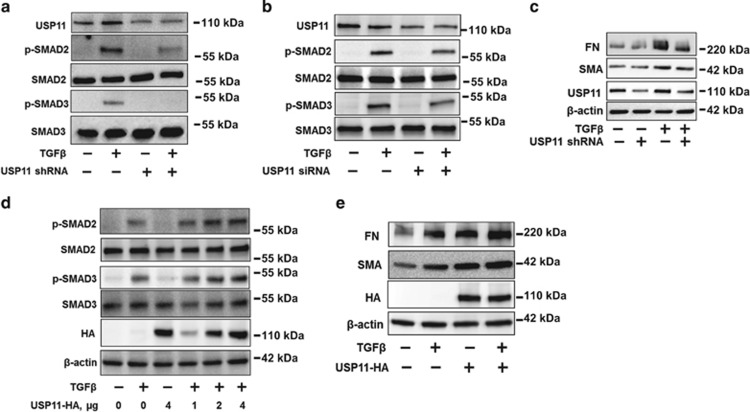
USP11 regulates TGF*β*-1 signaling pathway. (**a**) MRC5 cells were infected with cont shRNA (−) or USP11 shRNA lentivirus for 3 days as described in the Materials and Methods section, and then cells were treated with TGF*β*-1 (2 ng/ml) for 30 min. USP11 and phosphorylated and total SMAD2/3 levels were analyzed by western blotting. (**b**) MRC5 cells were transfected with cont siRNA (−) or USP11 siRNA for 3 days, and then cells were treated with TGF*β*-1 (2 ng/ml) for 30 min. USP11, total, and phosphorylated SMAD2/3 levels were analyzed by western blotting. (**c**) MRC5 cells were infected with cont shRNA (−) or USP11 shRNA lentivirus for 3 days, and then cells were treated with TGF*β*-1 (2 ng/ml) for 20 h. USP11, FN, SMA, and *β*-actin protein levels were analyzed by western blotting. (**d**) MRC5 cells were transfected with USP11-HA plasmids (0–4 *μ*g) for 2 days, and then cells were treated with TGF*β*-1 (2 ng/ml) for 30 min. USP11-HA, phosphorylated and total SMAD2/3, and *β*-actin levels were analyzed by western blotting. (**e**) MRC5 cells were transfected with USP11-HA plasmids (2 *μ*g) for 2 days, and then cells were treated with TGF*β*-1 (2 ng/ml) for 20 h. USP11-HA, FN, SMA, and *β*-actin protein levels were analyzed by western blotting. Western blotting images were cropped to improve the conciseness of the data; samples derived from the same experiment and the blots were processed in parallel. Representative of experiments performed at least three independent times

A recent study has revealed that MTX inhibits USP11 activity.^23^ To investigate the role of USP11 in TGF*β*-1 signaling in lung fibroblast cells, MRC5 cells were infected with USP11 shRNA lentivirus or transfected with USP siRNA. Both USP11 shRNA and USP11 siRNA resulted in the reduction of USP11 levels as well as diminishing p-SMAD2/3 levels, while maintaining total SMAD2/3 levels (Figures 2a and b). The downstream effects of TGF*β*-1 signaling were also examined by analyzing the expression levels of FN and SMA. As expected, the levels of these proteins were decreased when the cells were infected with USP11 shRNA lentivirus (Figure 2c).

Next, we determined whether overexpression of USP11 with an HA tag (USP11-HA) in MRC5 cells would enhance the expression and activation of T*β*RII. In support of a role for USP11 as a regulator of TGFβ signaling, we found that increasing doses of USP11-HA-transfected cells increased TGF*β*-1-induced phosphorylation of SMAD2/3 (Figure 2d) and expression of FN and SMA (Figure 2e). Taken together with data from MTX experiment, these results suggest that USP11 regulates TGFβ-1 signaling.

### MTX reduces TβRII stability by increasing its poly-ubiquitination

**Figure 3:**
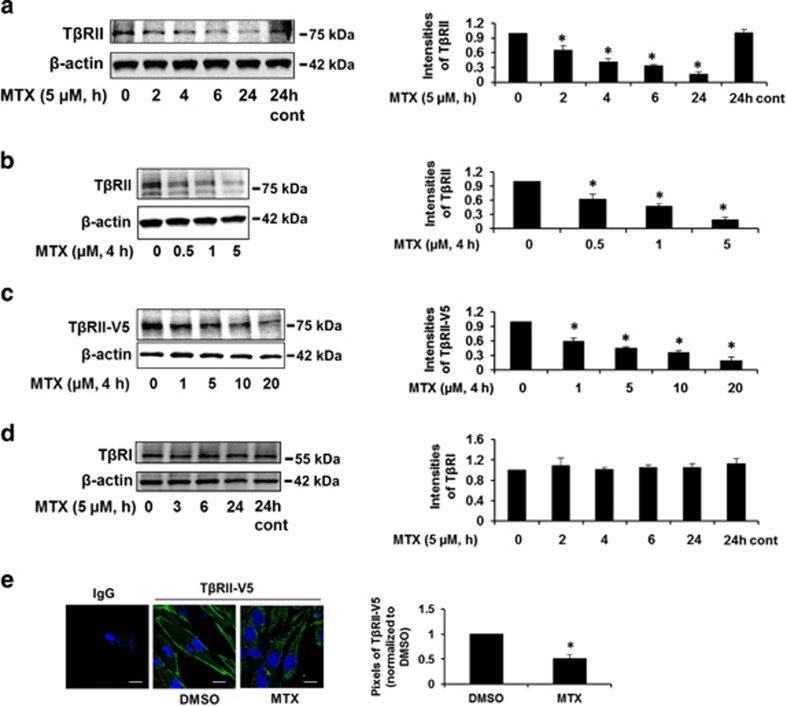
MTX reduces T*β*RII levels in human lung fibroblast cells. (**a**) MRC5 cells were treated with 5 uM MTX for 0–24 h. T*β*RII and *β*-actin levels were analyzed by western blotting. Intensities of T*β*RII were analyzed by the ImageJ software. *P<0.01 compared with 0 h. (**b**) MRC5 cells were treated with MTX (0–5 *μ*M) for 4 h. T*β*RII and *β*-actin levels were analyzed by western blotting. Intensities of T*β*RII were analyzed by the ImageJ software. *P<0.01 compared with 0 *μ*M. (**c**) MRC5 cells were transfected with T*β*RII-V5 plasmid for 48 h, and then cells were treated with MTX (0–20 *μ*M) for 4 h. T*β*RII-V5 and *β*-actin levels were examined by western blotting. Intensities of T*β*RII-V5 were analyzed by the ImageJ software. *P<0.01 compared with 0 *μ*M. (**d**) MRC5 cells were treated with 5 *μ*M MTX for 0–24 h. T*β*RI and *β*-actin levels were analyzed by western blotting. Intensities of T*β*RI were analyzed by the ImageJ software. Western blotting images were cropped to improve the conciseness of the data; samples derived from the same experiment and the blots were processed in parallel. Representative of experiments performed at least three independent times. (**e**) MRC5 cells grown on glass-bottom dishes were transfected with T*β*RII-V5 plasmid for 48 h, and then cells were treated with MTX (10 *μ*M) for 4 h. Localization of T*β*RII-V5 (green) in the cells were detected by immunostaining with V5 tag. DAPI (4,6-diamidino-2-phenylindole) was used for nuclei staining (blue). Bars, 50 *μ*m. Representative images were shown. Pixels of T*β*RII-V5 on the plasma membrane were quantified with the NIS-Elements software. *P<0.01 compared with dimethyl sulfoxide (DMSO)

To elucidate the molecular mechanisms by which MTX regulates TGF*β*-1 signaling, we examined the protein levels of T*β*RII. MTX reduced T*β*RII levels in a time- and dose-dependent manner (Figures 3a and b). Further, we investigated the effect of MTX on overexpressed T*β*RII. MRC5 cells were transfected with a V5-tagged T*β*RII (T*β*RII-V5) plasmid for 48 h. Cells were then treated with MTX for 4 h. As shown in Figure 3c, MTX reduced T*β*RII-V5 levels in a dose-dependent manner. Consistent with the data shown in Figure 1a, MTX had no effect on the levels of T*β*RI (Figure 3d). The reduction of T*β*RII by MTX was confirmed by immunofluorescence staining (Figure 3e), which indicate that MTX reduces T*β*RII levels in a short time frame (2–4 h).

**Figure 4:**
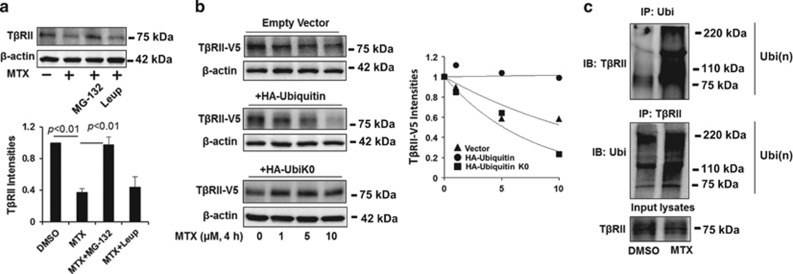
MTX promotes T*β*RII ubiquitination and degradation in the proteasome. (**a**) MRC5 cells were treated with MG-132 (20 *μ*M) or leupeptin (100 *μ*M) for 1 h prior to MTX treatment (5 *μ*M, 2 h). T*β*RII and *β*-actin levels were analyzed by western blotting. Intensities of T*β*RII were analyzed by the ImageJ software. (**b**) MRC5 cells were co-transfected with T*β*RII-V5 and with either empty vector, HA-ubiquitin, or HA-ubiquitin without lysine (HA-UbiK0) plasmids, cells were then treated with increasing concentrations of MTX (0–10 *μ*M) for 4 h. T*β*RII-V5 and *β*-actin levels were analyzed by western blotting. Intensities of T*β*RII-V5 were analyzed by the ImageJ software and then compared between the three groups. (**c**) MRC5 cells were treated with 5 *μ*M MTX for 1 h, and then cell lysates were subjected to immunoprecipitation with an ubiquitin antibody or a T*β*RII antibody, followed by T*β*RII or ubiquitin immunoblotting. Input lysates were analyzed by T*β*RII immunoblotting. Western blotting images were cropped to improve the conciseness of the data; samples derived from the same experiment and the blots were processed in parallel. Representative of experiments performed at least three independent times

To further investigate the molecular mechanisms by which MTX reduces T*β*RII levels, we focused on the effect of MTX on T*β*RII protein stability. Protein degradation occurs primarily through the proteasome or lysosome systems. MTX-induced T*β*RII degradation was inhibited by the proteasome inhibitor, MG-132, but not the lysosomal inhibitor, leupeptin (Figure 4a). This suggests that T*β*RII is degraded via the proteasome pathway. Therefore, we next explored the effects of overexpression of HA-tagged ubiquitin on MTX-induced T*β*RII degradation to reveal whether the T*β*RII proteasomal degradation is mediated by ubiquitination. As shown in Figure 4b, HA-ubiquitin overexpression promotes MTX-induced T*β*RII degradation, while overexpression of HA-ubiquitin lacking lysine residues (UbiK0), which losses its ability to form polyubiquitiantion, stabilizes T*β*RII. These observations support the conclusion that T*β*RII degradation by MTX is mediated by the ubiquitin–proteasome pathway. Additionally, MTX increased T*β*RII poly-ubiquitination, as indicated by the *in vivo* ubiquitination assay in Figure 4c.

### USP11 de-ubiquitinates and stabilizes T*β*RII

**Figure 5:**
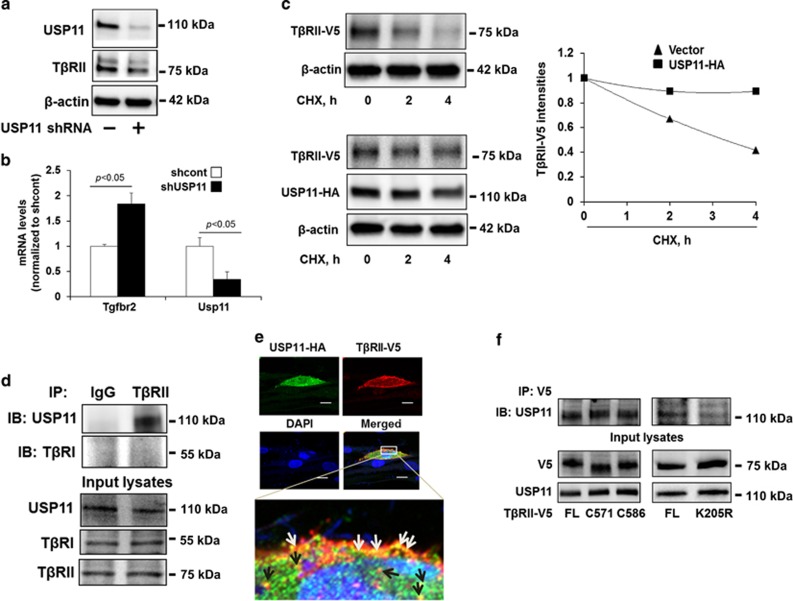
USP11 regulates T*β*RII stability in human lung fibroblast cells. (**a**) MRC5 cells were infected with cont shRNA (−) or USP11 shRNA lentivirus for 3 days, and then USP11, T*β*RII, and *β*-actin levels were analyzed by western blotting. (**b**) MRC5 cells were infected with cont shRNA (−) or USP11 shRNA lentivirus for 3 days, and then total RNA was extracted. Tgfbr2 and Usp11 gene expression were examined by RT-real time PCR. (**c**) MRC5 cells were co-transfected with T*β*RII-V5 and empty vector or USP11-HA plasmids for 48 h, and then cells were treated with CHX (20 *μ*g/ml) for 0–4 h. T*β*RII-V5, USP11-HA, and *β*-actin levels were analyzed by western blotting. Intensities of T*β*RII-V5 were analyzed by the ImageJ software and then compared between the two groups. (**d**) MRC5 cell lysates were subjected to immunoprecipitation with IgG or a T*β*RII antibody, followed by USP11 and T*β*RI immunoblotting. Input lysates were analyzed by USP11, T*β*RI, and T*β*RII immunoblotting. (**e**) MRC5 cells grown on glass-bottom dishes were co-transfected with USP11-HA and T*β*RII-V5 plasmids for 48 h. Localization of USP11-HA (green) and T*β*RII-V5 (red) in MRC5 cells were examined by immunostaining. Nuclei were stained by DAPI (4,6-diamidino-2-phenylindole; blue). USP11-HA and T*β*RII-V5 co-localization on the plasma membrane are indicated by white arrows; two protein co-localization in the cytoplasm are indicated by black arrows. Bars, 50 *μ*m. Representative images are shown. (**f**) MRC5 cells were transfected with plasmids encoding T*β*RII-V5 full-length (FL), C571, C586 deletion mutants, or K205R mutants for 48 h. Cell lysates were subjected to immunoprecipitation with a V5 antibody, followed by USP11 immunoblotting. Input lysates were analyzed by USP11 and V5 immunoblotting. Western blotting images were cropped to improve the conciseness of the data; samples derived from the same experiment and the blots were processed in parallel. Representative of experiments performed at least three independent times

To examine whether USP11 affects T*β*RII stability, MRC5 cells were infected with USP11 shRNA lentivirus, resulting in the reduction of both USP11 and T*β*RII protein levels (Figure 5a). To investigate whether this reduction occurred on the RNA or protein level, qPCR was performed to analyze mRNA levels for both USP11 and T*β*RII; this revealed that knockdown of USP11 has no reduction in the amount of RNA coded for T*β*RII (Tgfbr2) (Figure 5b). Therefore, the reduction of T*β*RII observed is on the protein level. Next T*β*RII protein stability was then examined by cycloheximide (CHX) chase assay, revealing the half-life of T*β*RII-V5 to be approximately 2 h. Overexpression of USP11-HA enhanced T*β*RII's lifespan (Figure 5c). Further, co-immunoprecipitation (Co-IP) confirmed that, in untreated lung fibroblast cells, as expected, T*β*RII does not interact with T*β*RI, while intriguingly, T*β*RII is associated with USP11 (Figure 5d). Both T*β*RII-V5 and USP11-HA are co-localized at the plasma membrane and cytoplasm in MRC5 cells (Figure 5e). To determine the USP11-binding site within T*β*RII, plasmids encoding two c-terminal deletion mutants (C571 and C586) and a K205R mutant of T*β*RII were generated. Co-IP experiments show that both C571 and C586 deletion mutants and wild type are associated with USP11, while T*β*RIIK205R exhibits a reduction of association with USP11 (Figure 5f), indicating that lys205 has a critical role in binding to USP11. Taken together, these results suggest that USP11 interacts and stabilizes T*β*RII.

**Figure 6:**
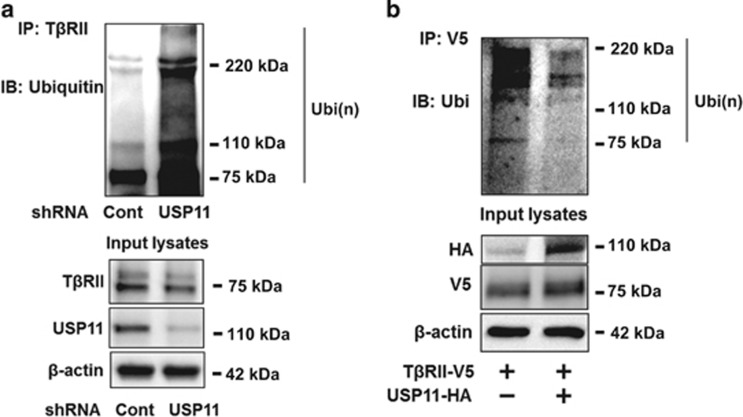
USP11 de-ubiquitinates T*β*RII. (**a**) MRC5 cells were infected with cont shRNA or USP11 shRNA lentivirus for 3 days. Cell lysates were subjected to immunoprecipitation with a T*β*RII antibody, followed by ubiquitin immunoblotting. Input lysates were analyzed by T*β*RII, USP11, and *β*-actin immunoblotting. (**b**) MRC5 cells were co-transfected with T*β*RII-V5 and empty vector or USP11-HA plasmids for 48 h, and then cell lysates were subjected to immunoprecipitation with a V5 antibody, followed by ubiquitin immunoblotting. Input lysates were analyzed by HA, V5, and *β*-actin immunoblotting. Western blotting images were cropped to improve the conciseness of the data; samples derived from the same experiment and the blots were processed in parallel. Representative of experiments performed at least three independent times

To investigate whether USP11 promotes T*β*RII stability through de-ubiquitinating T*β*RII, USP11 was downregulated by USP11 shRNA lentivirus infection prior to *in vivo* ubiquitination assay. As shown in Figure 6a, USP11 shRNA increased T*β*RII ubiquitination, whereas the level of ubiquitination was reduced by overexpression of USP11-HA (Figure 6b). Taken together, the data indicate that USP11 stabilizes T*β*RII through de-ubiquitinating T*β*RII. Inhibition of USP11 promotes T*β*RII proteasomal degradation, suggesting that USP11 is a pro-fibrotic DUB.

### USP11 and T*β*RII levels are increased in lung tissues from bleomycin-challenged mice and idiopathic pulmonary fibrosis (IPF) patients

**Figure 7:**
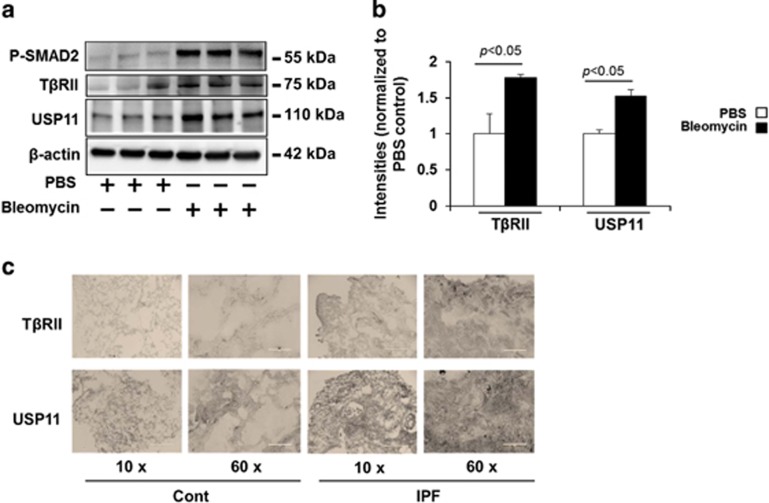
T*β*RII and USP11 are increased in the lungs from bleomycin-induced fibrosis model and IPF patients (**a**) C57BL/6 mice were challenged with intranasal injection of bleomycin for 3 weeks. P-SMAD2, T*β*RII, USP11, and *β*-actin levels were analyzed by western blotting. (**b**) Intensities of T*β*RII and USP11 were analyzed by the ImageJ software. (**c**) Human normal and IPF lung tissues were fixed and immunostained with T*β*RII and USP11 antibodies. Scale bars in × 10 image, 400 *μ*m; scale bars in × 60 images: 50 *μ*m. Representative images (from three per each group) are shown

Bleomycin-induced pulmonary fibrosis in mice is the most common experimental study model of human lung fibrosis. Phosphorylation of SMAD2 was detected in 3-week bleomycin-challenged mice, suggesting that TGF*β* signaling contributes to the pathogenesis of bleomycin-induced pulmonary fibrosis (Figure 7a). Both USP11 and T*β*RII were significantly increased in murine lung tissue samples from bleomycin-challenged mice (Figures 7a and b). Further, the expression levels of T*β*RII and USP11 in the lungs from normal and IPF patients were examined by immunohistochemistry. Both T*β*RII and USP11 are highly expressed in lung tissues from IPF patients (Figure 7c). It appears that both T*β*RII and USP11 are upregulated in most lung cells, including epithelial and fibroblast cells. These data suggest that USP11 may contribute to the pathogenesis of lung fibrosis through stabilization of T*β*RII and enhancement of TGF*β*-1 signaling in lung fibroblast cells.

## Discussion

**Figure 8:**
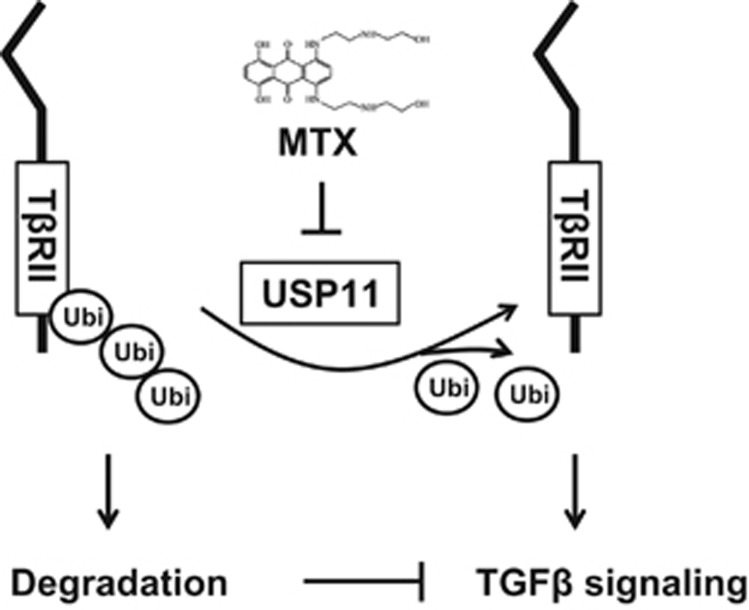
USP11 de-ubiquitinates and stabilizes T*β*RII. T*β*RII stability is regulated by its poly-ubiquitination. Destabilization of T*β*RII attenuates TGF*β*-1 signaling. USP11 stabilizes T*β*RII through de-ubiquitination of T*β*RII. MTX, an inhibitor of USP11, promotes ubiquitination and degradation of T*β*RII, thus mitigating TGF*β*-1 signaling

The TGF*β*-1 signaling pathway contributes in large part to the pathogenesis of fibrosis through its receptor T*β*RII/T*β*RI heterozygous complex.^25, 26, 27, 28^ Many studies have focused on examining the regulation of T*β*RI and its role in the progression of fibrosis.^8, 9, 10, 11, 20, 21^ However, very few studies have explored its co-receptor, T*β*RII. Similarly, numerous studies have illuminated the roles of DUBs in the regulation of T*β*RI signaling, including that of the role of USP11 in stabilization of T*β*RI.^21^ In the current study, we demonstrate that T*β*RII degradation is mediated by the ubiquitin–proteasome system. We found that USP11 stabilizes T*β*RII, not T*β*RI, in human lung fibroblast cells. Inhibition or knockdown of USP11 increases poly-ubiquitination of T*β*RII, thereby reducing T*β*RII stability and impairing TGF*β*-1 signaling in lung fibroblast cells (Figure 8). Further, we show that both USP11 and T*β*RII are highly expressed in murine lung tissues from experimental lung fibrosis and IPF patients. This study is the first to characterize the molecular regulation of T*β*RII stability by USP11 and suggests that USP11 is a potential target in treating fibrosis.

Ubiquitination is a reversible posttranslational modification process. DUBs counteract ubiquitination by removal of the ubiquitin chain from modified proteins. DUBs have key roles in the regulation of protein stability and signal transduction.^15, 16, 17, 19^ Several DUBs have been identified to regulate TGF*β*-1 signaling through targeting T*β*RI or SMADs. UCHL37, USP11, and USP15 de-ubiquitinate T*β*RI,^20, 21, 22^ while USP15, CYLD, and USP9X target SMAD4 or SMAD7.^29, 30, 31, 32^ In this study, we show that USP11 has no effect on T*β*RI stability in human lung fibroblast cells, though Al-Salihi *et al.*^21^ showed that USP11 de-ubiquitinates T*β*RI. This controversial conclusion may be due to use of different cell types. In their study, they investigated the effect of USP11 on T*β*RI de-ubiquitination in HEK293 cells, while we used human lung fibroblast cells. Consistent with their findings, we also revealed that USP11 promotes TGF*β*-1 signaling. In addition, we revealed that lys205 on T*β*RII is a USP11-binding site. Lys205 is considered one of potential ubiquitin-binding sites in T*β*RII according to a proteomic analysis.^33^ It is likely that an ubiquitin-like domain (UBL) in USP11 recognizes the ubiquitin-acceptor site on T*β*RII. To confirm this hypothesis, a UBL-deletion mutant of USP11 will be generated to test its role in interaction with T*β*RII. The novel finding in this study is that USP11 de-ubiquitinates and stabilizes T*β*RII. This is the first study to identify a DUB responsible for de-ubiquitination of T*β*RII. We demonstrate that knockdown of USP11 increases the ubiquitination of T*β*RII, whereas overexpression of USP11 greatly decreases ubiquitination of T*β*RII. This, in concordance with the results of the USP11 inhibitor treatment, proves that USP11 acts as a DUB, stabilizing T*β*RII and ultimately increasing the expression of profibrotic proteins, such as FN and SMA.

T*β*RII has been known to be posttranslationally modified by phosphorylation, ubiquitination, and neddylation.^34, 35, 36, 37, 38^ Autophosphorylation of T*β*RII is essential for its kinase activity and regulation of downstream signaling;^34^ however, the effect of phosphorylation of T*β*RII on its stability has not been revealed. Phosphorylation triggers receptor ubiquitination and internalization,^39, 40, 41^ therefore, it is possible that the phosphorylation of T*β*RII has a role in T*β*RII stability. Further studies will focus on investigating whether phosphorylation of T*β*RII affects USP11-mediated T*β*RII de-ubiquitination. Neddylation of T*β*RII by c-Cbl has been shown to antagonize T*β*RII ubiquitination and degradation.^38^ We found that MTX increases T*β*RII ubiquitination, while it reduces T*β*RII neddylation (data not shown), suggesting that the balance between ubiquitination and neddylation of T*β*RII is important for the regulation of T*β*RII lifespan and its pathophysiological effects. It will be interesting to study whether the phosphorylation determines the balance between ubiquitination and neddylation of T*β*RII.

As USP11 promotes TGF*β*-1 signaling through stabilization of T*β*RII, we hypothesize that USP11 may have a critical role in the development of lung fibrosis. Here we show that both USP11 and T*β*RII levels are increased in lung tissues from an experimental lung fibrosis model and IPF patients; however, the molecular regulation of USP11 has not been studied. We found that TGF*β*-1 has no effect on the expression of USP11, while it increases tyrosine phosphorylation of USP11 in lung fibroblast cells (data not shown). Taken together, targeting USP11 might be a new strategy to lessen the severity of lung fibrosis. MTX is an FDA-approved anticancer drug; the current study reveals that MTX antagonizes TGF*β*-1 signaling in lung fibroblast cells. Therefore, this study provides cellular and molecular evidence to indicate that MTX could be a potential antifibrotic drug to treat pulmonary fibrosis.

## Materials and Methods

### Cell culture and reagents

Human fetal lung fibroblast (MRC5) cell lines were propagated in EMEM media (Gibco, Rockville, IL, USA) with 10% FBS (Hyclone, Logan, UT, USA), and 1% penicillin/streptomycin antibiotic mix (Lonza, Allendale, NJ, USA). The cells were kept in a 37 °C humidified incubator with 5% carbon dioxide. Immobilized protein A/G beads, FN, α-SMA, T*β*RI, HA tag, USP11, V5 tag, T*β*RII, and control IgG antibodies, USP11 siRNA (pools of three to five siRNA), and control siRNA were from Santa Cruz Biotechnology (Santa Cruz, CA, USA). Phospho-SMAD2, phospho-SMAD3, total SMAD2, SMAD3, and ubiquitin antibodies were purchased from Cell Signaling Technology (Danvers, MA, USA). Bleomycin, leupeptin, CHX, MTX, and antibodies against Flag-tag and *β*-actin were from Sigma-Aldrich (St. Louis, MO, USA). Recombinant TGF-*β*1 was purchased from Invitrogen (Carlsbad, CA, USA). Proteasome inhibitor MG-132 was from Calbiochem (KGaA, Darmstadt, Germany). DAPI was purchased from ThermoFisher Scientific (Waltham, MA, USA). All materials in highest grades used in the experiments are commercially available.

### Cell lysis and immunoblotting

Following the aforementioned cellular treatments, cells were washed with cold PBS. Cells were then collected and lysed in 120 *μ*l of lysis buffer, which consisted of 20 mM Tris HCl (pH 7.4), 150 mM NaCl, 2 mM EGTA, 5 mM beta-glycerophosphate, 1 mM MgCl_2_, 1% Trton X-100, 1 mM sodium orthovanadate, 10 *μ*g/ml protease inhibitors, 1 *μ*g/ml aprotinin, 1μg/ml leupeptin, and 1μg/ml pepstatin. The cell lysates were then sonicated on ice for 12 s, followed by centrifugation at 4 °C at 5000 r.p.m. for 10 min. Protein concentrations of the samples were then determined with use of a Bio-Rad Protein Assay Kit (Bio-Rad Laboratories, Inc., Hercules, CA, USA) using BSA as a standard. Samples were all equilibrated to 20 *μ*g and run on a 4–15% SDS-PAGE gel, transferred to a nitrocellulose membrane, and blocked in 5% nonfat biological grade powdered milk dissolved in 25 mM Tris HCl (pH 7.4), 137 mM NaCl, and 0.1% TWEEN20 (TBST) for 30 min. Blots were washed with TBST and incubated with primary antibody in 5% BSA with TBST for 1 h or overnight. The membranes were then washed three times at 10 min intervals with TBST prior to addition of secondary antibody for 1 h. Blots were developed with an Enhanced Chemiluminescence Detection Kit (ThermoFisher Scientific, Waltham, MA, USA) according to the manufacturer's instruction.

### Plasmid construction and transfection

C-terminal deletion mutants of T*β*RII were generated by PCR with a plasmid encoding human T*β*RII as a template. Primers for T*β*RIIC571 (amino acid 1–571) and T*β*RIIC586 (amino acid 1–586) were : T*β*RII forward: 5′-CACCATGGTCGGGGGCTGCTCAGGGGC-3′ T*β*RIIC571 reverse: 5′-GAGCCTGTCCAGATGCTCCAGCTC-3′ T*β*RIIC586 reverse: 5′GCCGTCTTCAGGAATCTTCTC-3′. PCR products were inserted into pcDNA3.1/V5-His-TOPO vector. T*β*RIIK205R-V5 plasmid was generated using the QuickChange II XL Site-Directed Mutagenesis Kit (Agilent Technologies, Santa Clara, CA, USA) based on T*β*RII-V5 plasmid. The primers are: forward: 5′-GAGCTTCGGCGTCCTGCCGGTTTCCCA-3′ reverse: 5′-TGGGAAACCGGCAGGACGCGGAAGCTC-3′. All the deletion and site-mutation mutants were confirmed by DNA sequencing. HA-Ubiquitin plasmid (Addgene plasmid no. 18712) was a gift from Edward Yeh. HA-Ubiquitin-K0 was a gift from Ted Dawson (Addgene plasmid no. 17603, Cambridge, MA, USA). MRC5 (human lung fibroblast cell line) cells were grown on glass-bottom dishes, six-well plates, D100 and D60 dishes to 60–70% confluence. The cells were then transfected with varying amounts of plasmid using the Polyjet *In vitro* DNA Transfection Reagent (SignaGen Laboratories, Inc., Rockville, MD, USA) system based on the manufacturer's protocol.

### Co-IP and ubiquitination assay

MRC5 cells were cultured and transfected using the aforementioned protocols and then treated as indicated. The cells were then collected and lysates were prepared. Following the protein assay, lysate protein sample concentrations were equilibrated, input samples were removed, and antibody was added for overnight at 4 °C while rotating. The following morning, agarose beads were added and incubated in the cold on a rotator for at least 2 h. The samples were then centrifuged, supernatant removed, and the beads were washed for three times in PBS. In all, 2 × *β*-ME dye was added to the beads and heated at 100 °C for 10 min, and then the beads were removed by centrifugation. Samples were loaded on SDS-PAGE gels.

Cells were washed and collected with cold PBS for the ubiquitination assay. The cells in PBS were then centrifuged at 4 °C at 2000 r.p.m. for 5 min, the supernatant was removed, and 1 *μ*l of ubiquitin aldehyde and 1 *μ*l of NEM were added to the pellet. Based on the size of the pellet, 50–80 *μ*l of 2% SDS lysis buffer was added. The cells were then boiled at 100 °C for 10 min following sonication. After boiling, the sample was diluted with between 500 and 800 *μ*l of 1 × TBS. Normal IP procedure was then followed.

### Immunofluorescence staining

MRC5 cells were grown to 80–90% confluence on glass-bottom dishes and were treated as indicated. Following treatment, cells were washed with PBS, and fixed with 3.7% formaldehyde for 20 min. Cells were then washed three times with PBS, blocked in 1% BSA in TBST for 30 min, and washed three more times in PBS. Primary antibody was then incubated for 1 h. The cells were washed three times with PBS and incubated for 1 h in the dark with a fluorescent probe conjugated to the secondary antibody. DAPI was used to stain nuclei. Images were taken with a Nikon ECLIPSE TE 300 inverted microscope (Nikon, Tokyo, Japan).

### RNA isolation, reverse transcription, and qPCR

Total RNA was isolated from cells using the NucleoSpin RNA Extraction Kit from Clontech Laboratories, Inc (Mountain View, CA, USA). according to the manufacturer's protocol, and the isolated RNA was quantified using spectrophotometry. cDNA was then created using the iScript cDNA Synthesis Kit from Bio-Rad, per their specifications. mRNA expression levels of genes of interest were then analyzed by quantitative PCR using iQ SYBR Green Supermix and the iCycler Real-Time Detection System from Bio-Rad.

### Lentivirus preparation and infection

USP11 shRNA lentiviral vector plasmid encoding USP11-specific nucleotide shRNA (CCGGCCCTCCCTTCTAGTCTTTATTCTCGAGAATAAAGACTAGAAGGGAGGGTTTT) was obtained from Sigma-Aldrich. A HEK293T cell line and Lenti-X Lentivirus Packaging System (Clontech Laboratories, Inc.) were used to propagate the lentivirus used in the knockdown experiments. The manufacturer's protocol was followed. For each experiment using USP11 shRNA, 4 *μ*l of lentivirus was mixed with 1 *μ*l of hexadimethrine bromide (10 mg/ml) and added directly to the cells. Cells were then collected 72 h following inoculation.

### Bleomycin-induced murine model of pulmonary fibrosis

C57BL/6 mice with body weight of 20–25 g were purchased from the Jackson Laboratory (Bar Harbor, ME, USA). Bleomycin (0.045 U) was administered by intranasal injection. Partial right lungs were homogenized in cell lysis buffer. Protein levels were analyzed by western blotting using the indicated antibodies. Immunochemistry stainings were performed by pathology core facility at the University of Pittsburgh, Pittsburgh, PA, USA. All animal procedures in this study were performed in adherence with the National Institute of Health Guidelines on the use of Laboratory Animals and have been approved by the Institutional Animal Care and Use Committee of the University of Pittsburgh.

### Statistical analysis

All results were subjected to statistical analysis using Microsoft Excel (Microsoft, Redmond, WA, USA) or ANOVA, and wherever appropriate, the data were analyzed by Student's *t*-test and expressed as means±S.D. Data were collected from at least three independent experiments, and *P*<0.05 was considered significant.
